# A model for assessing the urban heat Island effect in urban regeneration areas: case of mamak and the north ankara

**DOI:** 10.1007/s00484-025-02908-5

**Published:** 2025-04-04

**Authors:** Mehtap Ozenen Kavlak, Muzeyyen Anil Senyel Kurkcuoglu, Alper Cabuk, Saye Nihan Cabuk, Mehmet Cetin

**Affiliations:** 1 Faculty of Open and Distance Education, Department of Geography, Istanbul University, Sariyer, İstanbul 34398 Türkiye; 2 Faculty of Architecture, Department of City and Regional Planning, Middle East Technical University, Ankara, 06800 Türkiye; 3 Faculty of Architecture and Design, Department of Architecture, Eskişehir Technical University, Eskisehir, 26555 Türkiye; 4 TAPLAK, Design and Planning Accreditation Association, Eskişehir, Türkiye; 5 Earth and Space Sciences Institute, Department of Geodesy and Geographical Information Technologies, Eskişehir Technical University, Eskisehir, 26555 Türkiye; 6 Faculty of Architecture, Department of City and Regional Planning, Ondokuz Mayis University, Samsun, 55270 Türkiye

**Keywords:** RS, UHI, Urban regeneration, Ankara

## Abstract

Urban regeneration, which aims to provide increased sustainability in terms of higher quality urban environment and better urban livability, has been on the agenda in Türkiye. Ankara, the capital city, has long faced uncontrolled development of squatter areas due to rural-to-urban migration, resulting in critical structural problems, including low-quality constructions, poor infrastructure, and insufficient urban services. Urban regeneration has been presented as a solution to those problems. This study investigates whether urban regeneration provides environmental benefits in terms of mitigating urban heat islands (UHI). Two large-scale urban regeneration areas in Ankara, the New Mamak Urban Regeneration Project (NMURP) and the North Ankara Urban Regeneration Project (NAURP), are anaylzed, both of which have been undergoing transformation more than a decade. Landsat 5 and Landsat 8 satellite images were used to detect the land use-based changes in the surface UHI, based on UHIER index, between 2005 and 2022, CORINE datasets were utilized for land use classification in the study areas for comparison. The results show that UHI values decreased in entire project areas due to removal of squatter settlements and partial completion of the transformation. However, when the local variations are observed, it is concluded that UHI values increased in already transformed sites as a result of high built-up densities, where complex cultivation pattern is replaced by urban fabric in NMURP, agriculture and construction sites replaced by urban fabric, natural grasslands replaced by road network and urban fabric, and urban fabric is replaced by urban green areas and construction sites in NAURP.

**Supplementary Information:**

The online version contains supplementary material available at 10.1007/s00484-025-02908-5.

## Introduction

Urban regeneration is associated with wide-ranging urban restructuring activities, including a holistic policy intervention incorporating physical, social, and environmental regeneration in declined and risky urban areas (Akkar 2006; Lang 2005; Richards 2014). Environmental sustainability is an indispensable component of urban regeneration, becoming increasingly prominent in international cases (Korkmaz and Balaban 2020; Xiuli and Maliene 2021). The environmental indicators of sustainability in regeneration activities include but are not limited to waste disposal and recycling, the use of renewable energy, building performance, housing layout and design, infrastructure improvements, pollution controls, density levels, green and open space, conservation, and public transportation opportunities (Aladağ and Işık 2016; Hemphill et al. 2004; Peng et al. 2015; Xiuli and Maliene 2021; Zheng et al. 2017). However, the primary motives and scope of the urban regeneration projects in Türkiye differ from those of their international counterparts. The contribution of regeneration practices to sustainability is often underestimated in the country (C. Korkmaz and Balaban 2020), and urban regeneration has been adopted as a form of transformation regarding project-based housing supply instead of a holistic process of which effects should be evaluated at the urban scale (Güzey 2009). Even more, the studies in the literature analyzing Turkish examples tend to overlook sustainability-oriented environmental impacts and focus on political, social, and economic repercussions in general. A limited number of studies investigate environmental impacts, yet, quantifiable environmental assessments, such as the Urban Heat Island (UHI), have not gotten enough attention.

Urban regeneration has been initiated as a state policy(Güzey 2009; Unsal 2015) aiming to produce more livable and sustainable living environments with better socio-economic opportunities and a well-protected environment against the problems arising from poor quality building stock, inadequate and outdated infrastructure, environmental degradation, and socio-economic integration problems of risky and degraded urban areas as well as squatter settlements. While the objectives of the regeneration activities are quite ambitious, the results, particularly the ones regarding environmental impacts, have remained vague and open to debate. Sustainability-oriented discussions take place in the literature, which evaluates the projects from a broader perspective, including environmental, social, and economic aspects, mainly through qualitative assessment methods (Eren and Tökmeci 2012; Kocabas 2005; Korkmaz and Balaban 2020; Uzun and Celik Simsek 2015).

Urban regeneration has several impacts both at the local and urban scales. The quantifiable environmental determinants are important to calculate for a solid discussion on these impacts. The UHI is among the significant reasons for increasing energy demand and pollution, which reduces air quality (Chun and Guldmann 2014; Grimm et al. 2008; Lowe 2016). Many researchers also associate critical health issues with the consequences of the UHI effect (Heaviside et al. 2017; Venter et al. 2020; Zhao et al. 2014). Increase in mortality and morbidity and reduced outdoor thermal comfort in urban spaces, directly impacting people’s quality of life and health, are also the most visible impacts of the UHI (da Silva Espinoza et al. 2023). Considering the urbanization and urban population growth projections, the cities will soon be exposed to even higher temperatures (Wang et al. 2019; Zoran 2011). UHI effect is considered higher in urban areas as land surface temperatures are also higher in cities compared to the rural (Tsou et al. 2017). Therefore, urban regeneration practices ignoring the relationship between the regenerated urban fabric and the UHI effect are likely to be accompanied by diverse negative consequences in the future. For example, impervious urban surfaces (Tian et al. 2021), heat-absorbing artificial materials, anthropogenic heat release, air pollution, tall and densely placed urban buildings disturbing ventilation, and inadequate vegetation cover (Aleksandrowicz et al. 2017; Gober et al. 2010; He 2019; Hu et al. 2021; Mirzaei 2015; Palme et al. 2017; Rizwan et al. 2008; Steeneveld et al. 2011) will most likely result in increased LST and UHI effects in the urban areas.

This paper aims to evaluate the environmental impact of urban regeneration projects in squatter settlements with a particular focus on the change in land surface temperatures (LST) and the surface UHI. The analysis not only evaluates the success of the projects but also enables the improvement of regeneration activities by producing effective policies and strategies. Two major urban regeneration areas/projects in Ankara, the New Mamak Urban Regeneration Project (NMRUP) and the North Ankara Urban Regeneration Project (NAURP), were selected as the study areas. Landsat 5 and 8 images were used to detect the land-use-based surface UHI changes between 2005 and 2022. Previous studies focused on the impacts of urban regeneration on quality of life, urban identity, socio-economic structure, and employment, as well as on current planning, implementation processes, and legislation. However, few studies analyzed the environmental impacts of urban regeneration practices from a perspective similar to that of this study. The novelty of this study lies in evaluating the impact of urban regeneration on UHI, which has received limited attention in the context of Ankara and Türkiye. Furthermore, the research gap that this study addresses is the lack of empirical evidence on the effectiveness of urban regeneration in mitigating UHI. The results of this study can be used to inform urban planning and development policies aimed at reducing the impact of UHI in urban areas.

## Materials and methods

### Study area

**Fig. 1:**
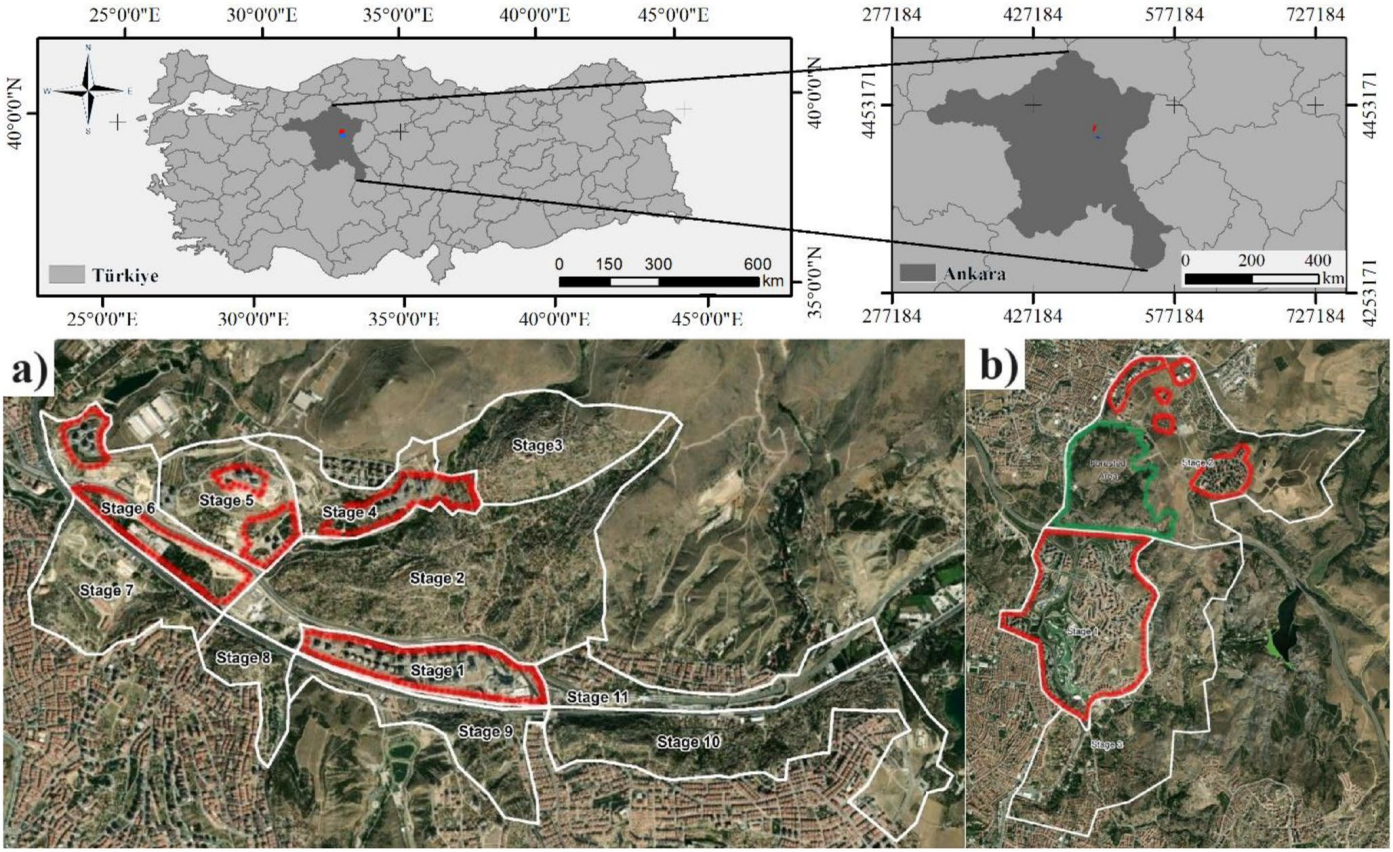
Study area (**a**) NMURP project area stages (**b**) NAURP project area stages (red areas indicate already regenerated areas)

The study area covers two large urban regeneration regions in Ankara: NMRUP and NAURP. These are among the largest urban regeneration areas in Türkiye, covering approximately 700 ha and 1583 ha, respectively. Both are located in the northeastern part of the city (Fig. 1). NAURP has 3 stages of implementation according to the master plan prepared by Ankara Metropolitan Municipality in 2011. The 1st stage comprises the priority transformation area (Korkmaz 2015), which has already been completed. NMRUP covers 11 stages. The clearance of squatters has almost been finalized for the whole project territory, and redevelopment of the 1st stage and partly the 4th, 5^th,^ and 6th stages are completed.

In NAURP, the emphasis was on the physical image, beautification, better environmental conditions, and construction of large green spaces (Tuç 2018).Demirtaş (2019) analyzed the NAURP in terms of urban ecology and concluded that the project did not consider renewable energy and climatic data in general, but the inclusion of landscape elements such as ponds and large green areas has been expected to reduce the UHI.Korkmaz and Balaban (2020) observed that the project offered limited environmental benefits due to poorly organized public transportation, car dependency, and the use of fossil fuels. However, they also concluded that the project provided a considerable amount of green spaces, a positive outcome of NAURP.

The aims of NMURP include the clearance of squatters just like the NAURP, overcoming the rough topographic conditions and the flooding risk of Hatip Stream, and increasing the area’s land rant (Gözözkut 2016). The project depends on the spatial arrangement in terms of the replacement of mostly single-storey squatters with high-rise blocks, which tend to increase residential densities in a significant way. Besides providing housing to squatter owners, new housing for 50,000 more residences is expected to financially compensate for the urban services, transportation, and landscape requirements (Somalı 2020).

### Data

Considering projects’ initiation and completion dates, Landsat 5 (TM) 2005 images (03.07.2005) were used to evaluate the status prior to the project implementations, and Landsat 8 2022 images (02.07.2022) were preferred for detecting the surface UHI for the post-regeneration process. The land use classes in the NMURP and NAURP regions were identified from the 2006 and 2018 CORINE Land Cover datasets, obtained from the Copernicus database. Landsat images were acquired from the USGS platform. Image quality, blur, and availability were evaluated for selecting the appropriate images. The resolution of the thermal band of Landsat is 100 m. However, it is resampled and brought up to 30 m by the data provider before serving to the users.

Study area borders were obtained in vector data format. Images were processed with ArcGIS 10.8.2, and statistical analyses were performed with Microsoft Excel.

### Method

**Fig. 2:**
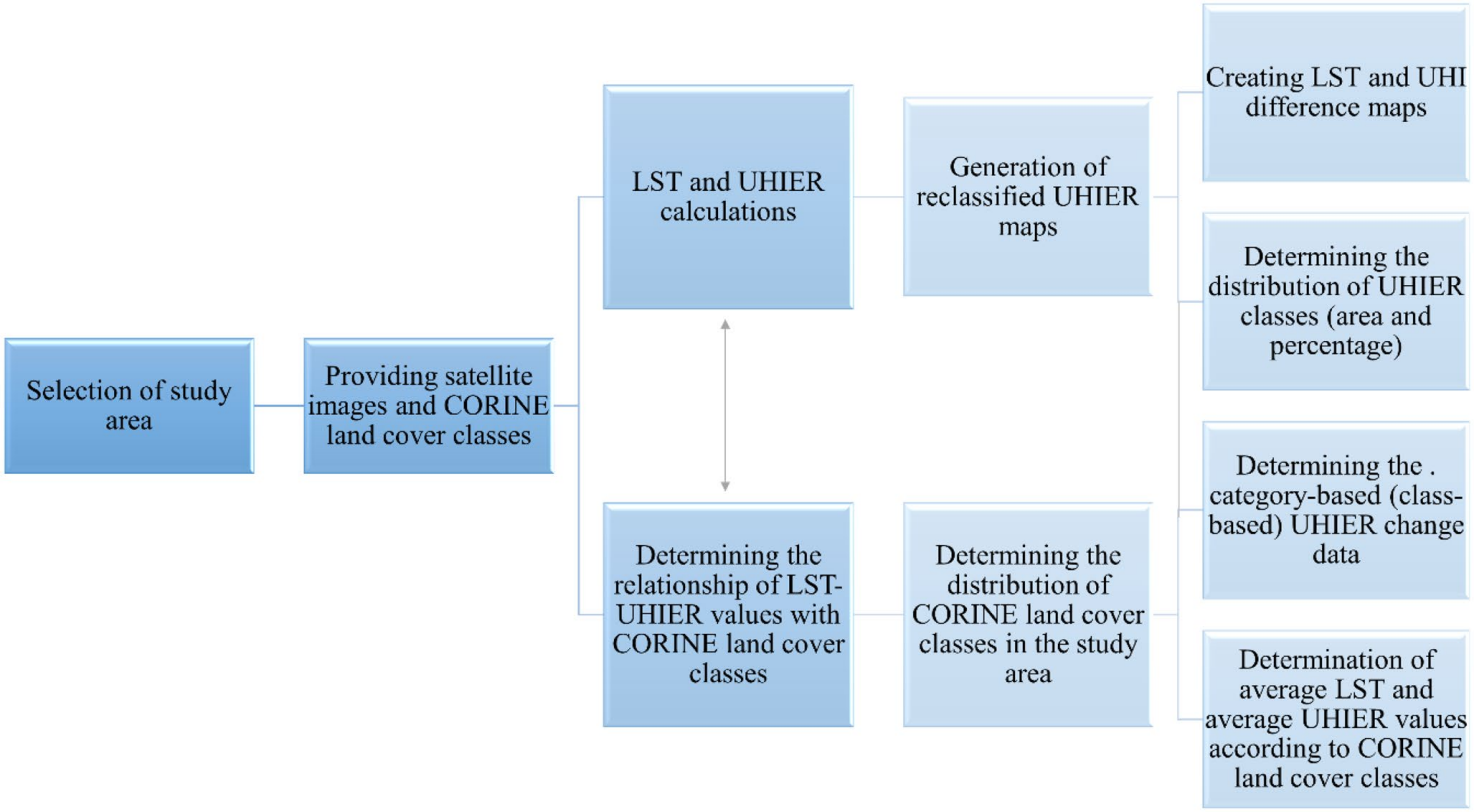
The workflow of the methodology

As LST-based UHI research deals with the temperatures at lower layers, Ayala-Carrillo et al. (2022) underline that this provides the determination of surface UHI. Since no meteorological observations are utilized in this study, the main approach is based on the determination of the LST for the study areas in 2005 and 2022, followed by UHIER calculations. The workflow of the methodology is presented in Fig. 2.

The thermal bands of Landsat are commonly used for UHI research (Halder et al. 2022; Hasanlou and Mostofi 2015; Seletković et al. 2023; Sheng et al. 2017). UHI effect research based on Landsat imagery requires applying a series of sequential processes, including NDVI, emissivity, and LST calculations. For better comprehension and discussion of the UHI effect, different approaches are adopted for evaluating the UHI intensity. This research applies the UHIER index to examine the quantitive differences in UHI between rural and urban lands. The primary index was created as UHI Ratio Index (URI) by Xu and Chen (2004), and was modified for other studies. The UHIER formula in this study is based on the research of Huang et al. (2019), explaining the UHIER as the relative LST examination. Appendix 1 summarizes the processes/formulas.

The UHIER values obtained by the application of the mentioned processes are usually classified into different categories considering the minimum and maximum values in the study areas (Huang et al. 2019; Zhang et al. 2013), each category referring to the amount of LST difference between the urban and rural areas. The negative values show the areas where urban land temperatures are lower than the rural and vice versa. Consequently, increasing negative values are associated with gradually decreasing LST values in urban areas compared to the rural, while increasing positive values show the degree of increased LST in the urban fabric. As the values get closer to 0, the difference between the compared areas decrease.

The UHIER values for the study areas in 2005 and 2022 were divided into 5 equal ranges: extremely low, low, medium, high, and very high. This classification approach also facilitates determining the increase and decrease rates in different land use types in the study area and associated locations.

UHIER difference maps were also developed using the raster calculator tool. Reclassified UHIER difference maps are essential as they help monitor the direction of the UHIER change. For example, visualization of a shift from high class in 2005 to low class in 2022 makes it possible to interpret the triggering reasons for this change regarding the target area’s geographical and land cover characteristics. Each pixel in the UHIER maps was assigned a point feature to create the attribute tables showing pixel UHIER values. Reclassified UHIER difference maps were divided into 5 difference categories ranging between high decrease (-2) and high increase (2) change classes.

The reference rural area in this study was determined following the approach adopted by Abbas and Hamdi (2022). Accordingly, two buffer rings of 5 km were created around the study areas, where the first zone was avoided due to the UHI footprint effect and the latter was used to determine the sample rural region. In the second buffer ring, mainly the north eastern regions were focused to avoid from the dense urban fabric patterns neighboring the southern and southwestern parts of the NMURP and NAURP boundaries, and the cloudy pixels in the satellite images. Lastly a 5 × 5 km sample area with rural land cover classes in CORINE dataset was determined.

Due to the risk for the 2018 CORINE data to accurately represent the actual land use classes in the study areas in 2022, a visual comparison was made. To determine the LST and UHIER values in each land use classes, and the pixel values were extracted to point feature class used for the statical analyses and calculations of the mean LST and UHIER values for each land use type.

## Results

### UHIER change in NMURP

**Fig. 3:**
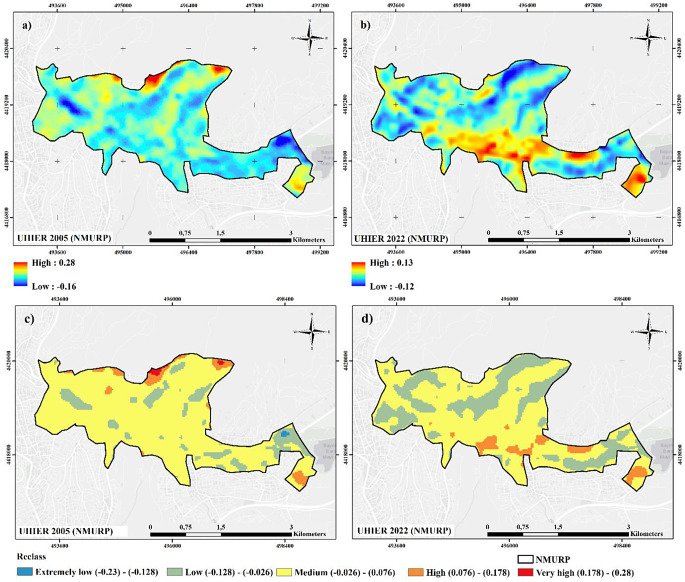
(**a**) 2005 UHIER map (**b**) 2022 UHIER map (**c**) 2005 reclassified UHIER map and (**d**) 2022 reclassified UHIER map for NMURP

Figure 3 illustrates the UHIER maps created for the NMURP region, and Table 1 gives information about the classification. The results show that the UHIER values ranged between − 0.16 and 0.28 in 2005 and − 0.12 and 0.13 in 2022, while reclassified maps reveal that high UHIER classes existed within the study area in 2005.

**Table 1:** Distribution of the UHIER classes (area and percentage) for NMURP

UHIER Value (Level ℃)	Level	Area (ha)		Area (%)	
		2005	2022	2005	2022
(-0.23) - (-0.128)	Extremely low	2	0	0	0
(-0.128) - (-0.026)	Low	100	240	13	30
(-0.026) - (0.076)	Medium	655	511	82	64
(0.076) - (0.178)	High	35	46	4	6
(0.178) - (0.28)	Very High	5	0	1	0

In line with the values in Table 1; Fig. 3 clearly present that the amount of low UHIER class increased in 2022, majorly transforming from medium UHIER category. In other words, the amount of cooler areas in the urban region increased compared to the average LST in the rural surrounding. However, the only extremely low region at the east territory, referring to the coolest LST degree, totally vanished in 2022. UHIER classes in already transformed areas, particularly in Stage 1, reflects high values in 2022, which replace medium and low values in 2005.

As also seen in Table 1, low and medium UHIER areas occupy 13% (100 ha) and 82% (655 ha) of the NMURP region in 2005. These rates for the same categories are 30% (240 ha) and 64% (511 ha) in 2022. High UHIER areas increased from 35 ha to 46 ha, and the very high class of 5 ha in 2005 vanished in 2022.

**Fig. 4:**
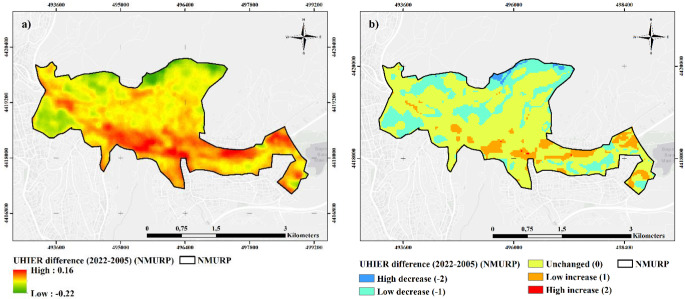
(**a**) Streched and (**b**) Reclassified UHIER difference maps for NMURP

Figure 4 shows the UHIER difference maps for the NMURP region between 2005 and 2022. The results indicate an increase in the south and southeast parts of the study area in 2002, while the UHIER values decrease in the north, west and northwest parts of the study area. As to the reclassified UHIER difference map (Fig. 4b), the highest decrease (-2) was observed in the northern territories and the highest increase (2) in the southern and south western regions.

According to Table 2, which shows the direction of the change in UHIER classes between 2005 and 2022, 100% of the extremely low UHIER areas in 2005 converted to low UHIER, and 78% of the high UHIER areas in 2005 became medium UHIER areas in 2022. Besides, all very high UHIER areas in 2005 were replaced with medium category in 2022, indicating a remarkable change.

**Table 2:** Category-based (class-based) UHIER change data for NMURP between 2005–2022

2005	2022	Area (ha)	Change
Extremely low	Low	2	Increase
Low	Low	69	No change
Low	Medium	30	Increase
Low	High	0	Increase
Medium	Low	166	Decrease
Medium	Medium	448	No change
Medium	High	41	Increase
High	Low	4	Decrease
High	Medium	28	Decrease
High	High	4	No change
Very high	Medium	5	Decrease

When the changes in UHIER classes are examined, there is a total 73 ha increase in contrast to 203 ha decrease in NMURP. Almost 521 ha area remained unchanged, meaning that the UHIER status of the previous period is continued. The most prominent decrease was observed in medium to low category with a 166 ha decrease, whereas the most significant increase has been realized in medium to high group with 41 ha increase. Although an overall decrease seems to be achieved, spatial variations of the UHI changes show that regenerated areas such as the urban developments in the 1st and the 6th stages resulted in UHI increase rather than decrease. Other regeneration areas’ UHIER values seem to remain unchanged, yet, it is not possible to argue that regeneration led to a decrease.

### UHIER change in NAURP

**Fig. 5:**
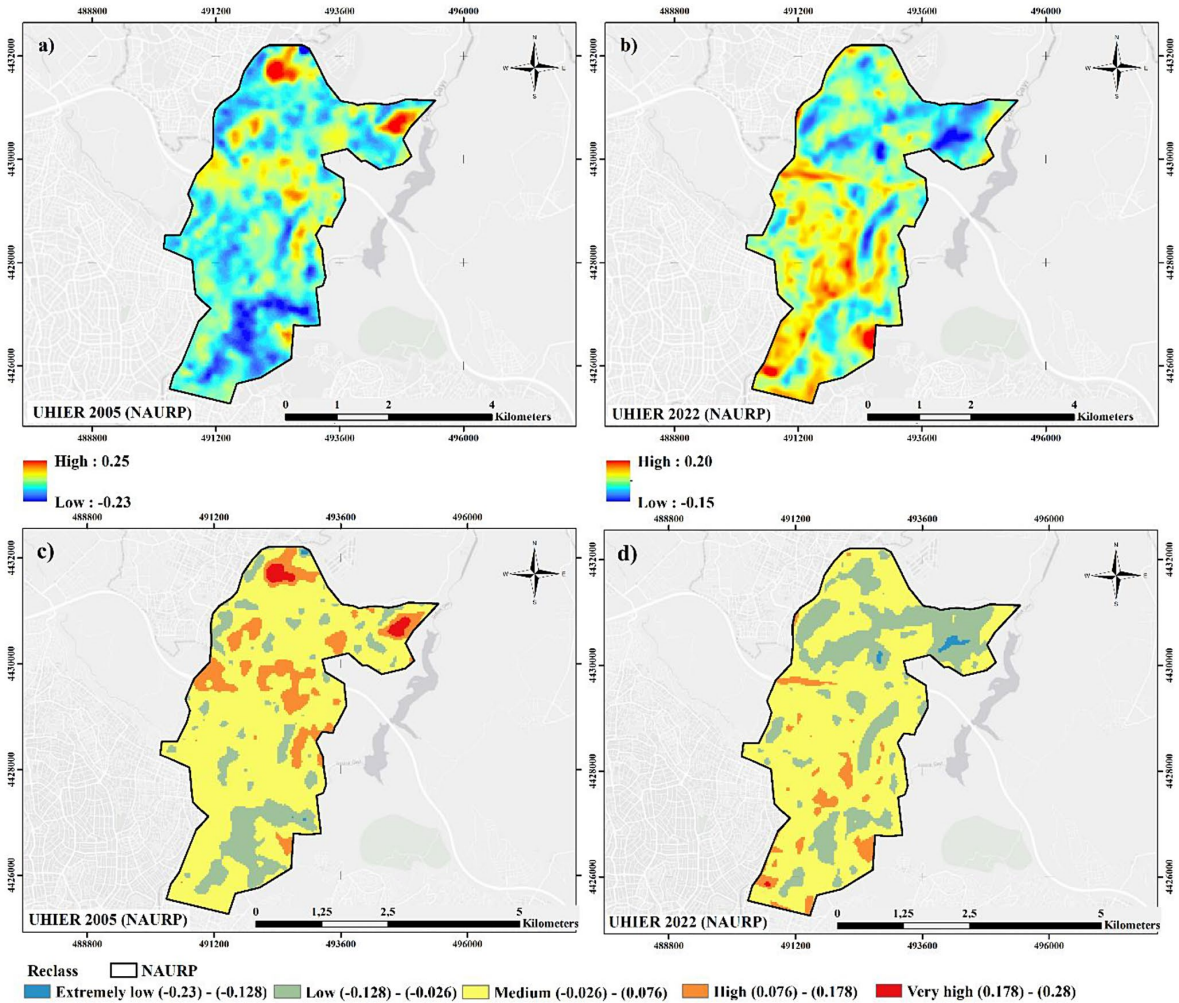
(**a**) 2005 UHIER map (**b**) 2022 UHIER map (**c**) 2005 reclassified UHIER map and (**d**) 2022 reclassified UHIER map for NAURP

**Fig. 6:**
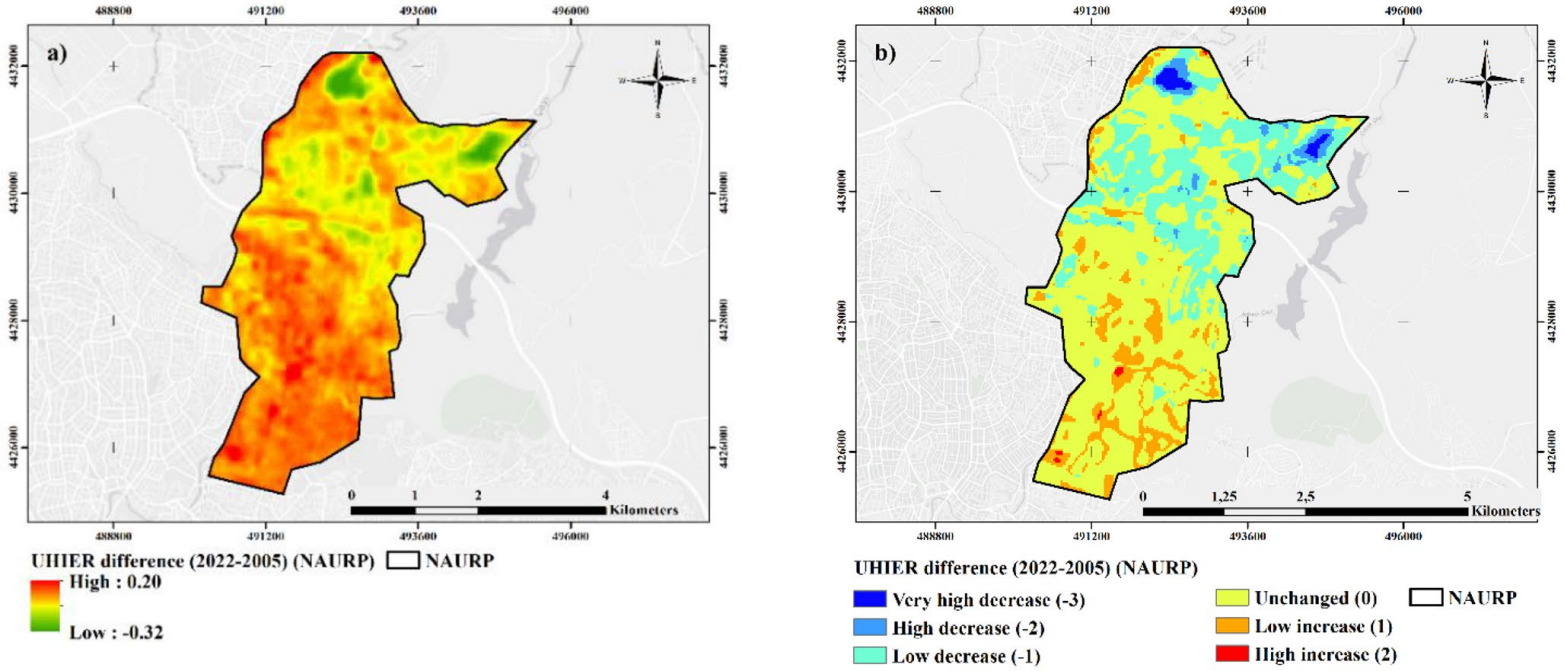
(**a**) Stretched and (**b**) Reclassified UHIER difference maps for NAURP

The calculation and analysis results for the NAURP region illustrated in Fig. 5 reveal that UHIER values varied between − 0.23 and 0.25 in 2005 and − 0.15 and 0.20 in 2022. Reclassified UHIER maps show that the high and extremely high UHIER areas in 2005 concentrated in the north half of the study area mostly disappeared in 2022, and though considerable small extremely low UHIER areas emerged in the northwest part. The study area dominantly possesses medium UHIER class, which refer that the LST values between the rural and the urban regions are close. According to the data in Table 3, the distribution of the low and medium UHIER areas was found to be approximately 16% (268 ha) and 71% (1195 ha) in 2005 and 27% and 67% in 2022, referring to a increase in the former and an decrease in the latter. High UHIER areas decreased to 87 ha, while the very high UHIER class of 1 ha totally disappeared in 2022. The remarkable increase in the low UHIER category is mostly from the high and very high class. Figure 6 depicts the UHIER difference maps for the NAURP region. The results show that the UHIER values in the southern half of the study area increased in 2022. In contrast, the northern parts of the NAURP region went through a decrease in terms of UHIER. When the reclassified UHIER differences are examined (Fig. 6b), the highest decrease (-3 and − 2) rates were mainly observed in the north and northwest regions of the study area. On the other hand, a high increase (2) was observed in the southern and central parts.

**Table 3:** Distribution of the UHIER classes (area and percentage) for NAURP

UHIER Value (Level ℃)	Level	Area (ha)		Area (%)	
		2005	2022	2005	2022
(-0.23) - (-0.128)	Extremely low	2	9	0	1
(-0.128) - (-0.026)	Low	268	455	16	27
(-0.026) - (0.076)	Medium	1195	1139	71	67
(0.076) - (0.178)	High	203	87	12	5
(0.178) - (0.28)	Very High	23	1	1	0

The changes in the UHIER classes between 2005 and 2022 in the NAURP region (Table 4) remarkably present that 79% of the extremely low UHIER areas in 2005 changed into low UHIER areas in 2022, while 80% of high UHIER areas in 2005 were transformed into mediım, and 75% of very high into low category. When the overall changes in UHIER groups are investigated, 184 ha of increase and 480 ha of decrease are calculated while 1027 ha remained unchaged in NAURP. Similar to the NMURP case, the prominent decrease is observed in medium to low, with 261 ha decrease. However, the highest inrease is realized in low to medium group, with 111 ha increase. The local variation of the increased and decreased areas are observed, regeneration sites reflect a distinctive pattern. Those areas, particularly the 1st stage, seems to either increase or remain unchanged, whereas the forested area in the second stage resulted in a decrease in general.

**Table 4:** Category-based (class-based) UHIER change data for NAURP between 2005–2022

2005	2022	Area (ha)	Change
Extremely low	Low	1	Increase
Extremely low	Medium	0	Increase
Low	Extremely low	3	Decrease
Low	Low	151	No change
Low	Medium	111	Increase
Low	High	3	Increase
Medium	Extremely low	6	Decrease
Medium	Low	261	Decrease
Medium	Medium	860	No change
Medium	High	68	Increase
Medium	Very high	1	Increase
High	Low	25	Decrease
High	Medium	162	Decrease
High	High	16	No change
Very high	Low	17	Decrease
Very high	Medium	6	Decrease

### Land use based LST and UHIER changes in NMURP ve NAURP regions

Table 5 summarizes the distribution of the 2006 and 2018 CORINE land use classes in NMURP and NAURP regions. 7 classes in NMURP and 11 classes in NAURP exist in 2006 and 2018 CORINE reveal 8 and 12 land use classes in the study areas respectively. The land use areas below 0.3 ha were ignored in the study areas. With a remarkable rate in NMURP (81% in 2006 and 91% in 2018), discontinous urban fabric has the highest occupation rate for both regions in 2006 and 2018. The amount of natural grasslands in NAURP region is also distinctive as they spread over 18% of the area in 2006 and 16% in 2018. The mean UHIER values in the project areas were recalculated for all land use types and the results are presented in Table 6. There is an increase in average UHIER values in continuous urban fabric and discontinuous urban fabric in NAURP, although the size of both land use classes decreased. Green urban lands appear to increase both in terms of size and UHIER values. However, it should be noted that the changes are not more than 2% in NAURP, except for an increase in green urban areas by 5%. In NMURP the values either decreased or remained unchanged. Since these values represent averages for the entire project area, the most intriguing results are in discontinuous urban fabric, while the UHIER value of this land class decreased slightly in contrast to the increase in the area by 10%. This might be due to the removal of the scattered squatter units and construction of more compact built-up areas.

**Table 5:** The distribution of CORINE land use classes in the study areas in 2006 and 2018

	NMURP				NAURP			
	2006	2018	2006	2018	2006	2018	2006	2018
	Area (ha)	Area (ha)	Percent (%)	Percent (%)	Area (ha)	Area (ha)	Percent (%)	Percent (%)
Continuous urban fabric	-	-	-	-	4	1	3	1
Discontinuous urban fabric	59	66	81	91	59	55	38	36
Industrial or commercial units	2	1	3	2	3	2	2	1
Road and rail networks and associated land	-	-	-	-	5	7	4	5
Construction sites	-	-	-	-	3	4	2	2
Green urban areas	-	-	-	-	-	8	-	5
Non-irrigated arable land	2	1	2	2	11	13	7	8
Pastures	-	-	-	-	8	4	5	3
Complex cultivation patterns	8	2	11	2	11	12	7	8
Land principally occupied by agriculture, with significant areas of natural vegetation	-	-	-	-	14	13	9	8
Natural grasslands	2	1	2	2	28	24	18	16
Transitional woodland-shrub	1	1	1	1	7	11	5	7

**Table 6:** Average UHIER (°C) values for the land use classes in the study areas

	NMURP		NAURP	
	UHIER 2005	UHIER 2022	UHIER 2005	UHIER 2022
Continuous urban fabric	-	-	0.02	0.06
Discontinuous urban fabric	0.01	0	0.01	0.03
Industrial or commercial units	0.05	-0.02	0.01	0.01
Road and rail networks and associated land	-	-	0.07	0.04
Construction sites	-	-	0.06	0.05
Green urban areas	-	-	-	0.01
Non-irrigated arable land	0.08	0.08	0.06	-0.03
Pastures	-	-	0.04	-0.01
Complex cultivation patterns	-0.01	-0.03	-0.06	-0.02
Land principally occupied by agriculture, with significant areas of natural vegetation	0.04	-0.01	0.04	-0.06
Natural grasslands	0.1	-0.02	0.03	-0.01
Transitional woodland-shrub	-0.02	-0.02	-0.01	-0.03

## Discussions

UHI is analyzed for two time periods covering before and after regeneration. To provide a concrete basis for a comparative analysis, the actual land use change has been retrieved from CORINE land cover data sets for the closest years possible. The land use distribution in 2006 is dominated by discontinued urban fabric and few complex cultivation patterns in NMURP. Almost all cultivation patterns seem to be converted to discontinuous urban fabric in 2018 with a 10% increase in discountinuous urban fabric and a 9% decrease in complex cultivation. Land use types in NAURP are more diverse, yet dominated by discontinuous urban fabric. One of the most significant change seems to be realized in urban regeneration areas where the construction sites and pastures turned into discontinuous urban fabric in the north-east side of the project area, in Stage 2. Another significant change has been realized in Stage 1 where urban discontinuous fabric is replaced by green urban areas and construction sites. Green urban areas experienced the greatest aerial increase with 8 ha, then 1 ha increase in construction sites. Almost 2 ha increase is observed in road and rail network from 2006 to 2018, which is plausible since the conversion of squatter areas to planned settlements.

The outputs show that UHI tends to decrease at the overall for both projects. 203 ha decrease is observed in NMURP in contrast to 73 ha increase among five classes. The highest decrease among UHIER groups is observed from medium to low category with a 166 ha decrease, whereas the highest increase is evident from medium to high group with a 41 ha increase. Previously high values either decreased or remained unchanged while previously low groups either increased or remained unchanged, indicating that the urban regeneration generally creates an upward effect in low UHI values. In NAURP, similar results were obtained, since UHI tends to decrease in the entire area with 480 ha of decrease in contrast to 184 ha of increase in UHIER groups in the entire area. The highest decrease is observed from medium to low group with 261 ha change, whereas the highest increase is observed from low to medium group with 111 ha change. High and very high UHI groups either decreased or remained unchanged. Very low values seem to be swept away between the two periods, again implying the upward effect of regeneration in low UHI categories, similar to NMURP.

At that point, changes in average values over the entire area might be misleading in terms of spatial variation of the results. Although the decreased UHI values are larger than increased UHI values in both areas, where these changes take place is a matter that needs to be evaluated. Mapping of UHI differences shows that the most significant UHI increases have been observed in already transformed areas. In NMURP, regenerated areas in Stage 1 and Stage 6 result in UHI increase. UHI decrease, on the other hand, is mostly observed in the northern part, where regeneration has not been started yet but most of the squatters have been removed. In NAURP, UHI increases in regenerated areas are more obvious, particularly in Stage 1. In spite of the implementation of a large urban green space, UHI values increased in this area. In addition to these, regenerated areas in Stage 2 and removed squatter areas besides the new construction sites in Stage 3 reflect higher UHI values in 2022 when compared to 2005. Decreased UHI values are primarily observed in the forested area and some agricultural patches in the north, as well as the hillsides in the eastern part of the project area. However, it is apperant that urban regeneration does not alleviate UHI, on the contrary, it had an intensifying impact. UHI changes in land use classes support the results, since average UHIER values increased in both continuous and discontinuous urban fabric, as well as green urban areas. In that sense, the results support the findings ofKorkmaz & Balaban (2020) stating that the project has limited environmental benefits, while contradicts with the comments of(Demirtaş 2019) assuming that large green areas would decrease UHI.

Both projects are ongoing and the remaining stages cover transformation from squatter to planned urban settlements with similar FARs. Thus, the risk of increased UHI should be considered, since such high building densities tend to increase surface temperatures in spite of large urban parks as in the case of NAURP. Future regeneration projects are also better take UHI effect into account to achieve higher environmental qualities than unauthorized settlements.

## Conclusions

In this research environmental impact of urban regeneration is examined in two different project areas in terms of the changes in surface UHI. One of the main motives of this study is providing guidance for future urban regeneration plans in terms of the UHI effect and serve as a model for similar studies in other urban areas facing similar urbanization problems. The study areas provide a good sample for comparison, as they include both areas that have already been regenerated and areas that regeneration has not started yet. Moreover, the projects locate on the periphery, thus, the landuse of the surrounding areas do not reflect a drastic change during the study period, which supports the robustness of the calculations.

The findings of this study have practical implications for policymakers and urban planners. It is observed that urban regeneration do not provide environmental benefits in mitigating UHI, even large urban green areas are provided. Therefore, future urban regeneration projects are better take into account the built-up densities and the amount of paved surfaces if they aim to create a better environmental quality and improved quality of life. Policymakers and urban planners could use the results of this study to develop evidence-based policies and strategies to mitigate UHI. Another contribution of the study is the use of RS techniques and analysis methods to monitor changes in the UHI over time.

There are some limitations of the study, as well. The study does not take the effect of the building shadows into account. In fact, the daytime satellite imagery is utilized for the summer season, which minimized the shadow effect and also when the meteorological conditions are more stable. The future timings of the projects are uncertain since there is not an officially agreed and announced schedule for the following stages and the process is affected from a complex combination of many factors, especially financing.

## Electronic supplementary material

Below is the link to the electronic supplementary material.

Supplementary Material 1

## Data Availability

The datasets used and/or analyzed during the current study are available from the corresponding author on reasonable request.

## Data availability

The data of the study will be provided upon request.
