# Supplementary material for: A model for assessing the urban heat Island effect in urban regeneration areas: case of mamak and the north ankara
Source: Int J Biometeorol. 2025 Apr 4;69(7):1517–28. doi: 10.1007/s00484-025-02908-5 (PMC12179009; doi:10.1007/s00484-025-02908-5)
Supplement: Supplementary file 1 — Supplementary Material 1 [file 484_2025_2908_MOESM1_ESM.docx]

**Appendix 1**

A. Analyses and formulas used for Landsat 5 data-based UHI research in the study

| **Analysis** | **Formula** | **Definitions** |
| --- | --- | --- |
| Normalized Difference Vegetation Index (NDVI)  (Cetin et al., 2022) | (𝑁𝐼𝑅 – 𝑅𝐸𝐷) / (𝑁𝐼𝑅 + 𝑅𝐸𝐷) | - NIR = 4^th^ band (Landsat 5) - RED = 3^rd^ band (Landsat 5) |
| Energy radiation (Ls)  (Walawender, 2009) | ((L_max_-L_min_) / 255) DN + L_min_ | - L_max_: Maximum value of energy radiation for each scene [W m^−2^ sr^−1^ μm^−1^] - L_min_: Minimal value of energy radiation for each scene [W m^−2^ sr^−1^ μm^−1^] - DN: Digital number (value of each pixel, band 6 thermal infrared) |
| Radiation temperature (brightness temperature) [K] (T_R_)  (Chander & Markham, 2003) | K_2_/(ln((K_1_/Ls)+1) | - Ls: Energy radiation [W m^−2^ sr^−1^ μm^−1^] - K_1_: Landsat 5 calibration constant = 607.76 [W m^−2^ sr^−1^ μm^−1^] - K_2_ : Landsat 5 calibration constant = 1260.56 [K] |
| Proportion of vegetation (P_V_)  (Kumar et al., 2022) | ((𝑁𝐷𝑉𝐼 − 𝑁𝐷𝑉𝐼_𝑚𝑖𝑛_)/ (𝑁𝐷𝑉𝐼_𝑚𝑎𝑥_ − 𝑁𝐷𝑉𝐼_𝑚𝑖𝑛_))^2^ | - The minimum and maximum values of the NDVI image can be displayed directly in the image |
| Land surface emissivity (ε)  (Kumar et al., 2022) | 0.004 ∗ 𝑃𝑣 +0.986 | - Pv = Proportion of vegetation - The value of 0.986 corresponds to a correction value of the Equation. |
| Land surface temperature [K] (LST_K_)  (Lillesand et al., n.d.) | T_R_/( ε)^0.25^ | - TR: Radiation temperature [K] - ε : Emissivity |
| LST [^o^C] | LST_K_ – 273.15 | - To obtain the results in Celsius, the radiant temperature is adjusted by adding the absolute zero (approx. −273.15 °C) |
| UHI intensity (UHIER)  (Huang et al., 2019) | ΔTi/Ts = (Ti-Ts)/Ts | - Ti = The LST of the i-th pixel - Ts = The mean LST of rural lands |

B. Analyses and formulas used for Landsat 8 data-based UHI research in the study

| **Analysis** | **Formula** | **Definitions** |
| --- | --- | --- |
| Normalized Difference Vegetation Index (NDVI)  (Cetin et al., 2022) | (𝑁𝐼𝑅 – 𝑅𝐸𝐷) / (𝑁𝐼𝑅 + 𝑅𝐸𝐷) | - NIR = 5^th^ band (Landsat 8) - RED = 4^th^ band (Landsat 8) |
| Top of atmospheric reflectance (TOA)  (Kumar et al., 2022) | 𝑀𝐿 ∗ 𝑄𝑐𝑎𝑙 + 𝐴L | - ML = Radiance multiplicative Band (No.10) - AL = Radiance Add Band (No.10) - Qcal = Quantized and calibrated standard product pixel values (DN) |
| TOA to brightness temperature (BT) conversion  (Kumar et al., 2022) | (𝐾_2_/(ln(𝐾1/𝐿)+ 1)) −273.15 | - K_1_ = Band-specific thermal conversion was constant from the metadata (774.8853) - K_2_ = Band-specific thermal conversion was constant from the metadata (1321.0789) - L = TOA - To obtain the results in Celsius, the radiant temperature is adjusted by adding the absolute zero (approx. −273.15 °C) |
| Proportion of vegetation (P_V_)  (Kumar et al., 2022) | ((𝑁𝐷𝑉𝐼 − 𝑁𝐷𝑉𝐼_𝑚𝑖𝑛_)/ (𝑁𝐷𝑉𝐼_𝑚𝑎𝑥_ − 𝑁𝐷𝑉𝐼_𝑚𝑖𝑛_))^2^ | - The minimum and maximum values of the NDVI image can be displayed directly in the image |
| Land surface emissivity (ε)  (Kumar et al., 2022) | 0.004 ∗ 𝑃𝑣 +0.986 | - Pv = Proportion of vegetation - The value of 0.986 corresponds to a correction value of the Equation. |
| LST  (Kumar et al., 2022) | (𝐵𝑇 / (1 + (λ∗ 𝐵𝑇 / C2) ∗ 𝐿𝑛(ε)))  (𝐵𝑇 / (1 + (0.00115 ∗ 𝐵𝑇 / 1.4388) ∗ 𝐿𝑛(ε))) | - BT = Top of atmosphere brightness temperature (°C) - λ = Wavelength of emitted radiance (0.00115) - C2= h*c/s = 1.4388×10–2 mK =14,388 mK   h= Planck's constant =6.626×10–34 J s  s= Boltzmann constant =1.38×10–23 JK  c= Velocity of light =2.998×108 m/s   - ε = Land surface emissivity |
| UHI intensity (UHIER)  (Huang et al., 2019) | ΔTi/Ts = (Ti-Ts)/Ts | - Ti = The LST of the i-th pixel - Ts = The mean LST of rural lands |
